# Primary Squamous Cell Carcinoma of Thyroid: A Rare Entity

**DOI:** 10.1155/2015/838079

**Published:** 2015-06-29

**Authors:** Rahulkumar N. Chavan, Bhargav Chikkala, Cinjini Biswas, Somak Biswas, Diptendra Kumar Sarkar

**Affiliations:** ^1^Department of General Surgery, IPGMER, Kolkata 700025, India; ^2^DY Patil University School of Medicine, Navi Mumbai, India; ^3^TMH, Kolkata, India; ^4^KEM Hospital, Mumbai, India

## Abstract

Primary squamous cell carcinoma (PSCC) of thyroid is an extremely rare malignancy of thyroid. Herewith, we describe a case report of female patient who presented with neck swelling; FNAC misdiagnosed it as papillary carcinoma of thyroid but, after resection, biopsy revealed it to be a case of squamous cell carcinoma of thyroid. After extensive investigations no possible primary focus of squamous cell carcinoma was found elsewhere, so diagnosis of primary squamous cell carcinoma of thyroid was made. Patient underwent chemoradiation but still patient succumbed to death within a year.

## 1. Introduction

Primary squamous cell carcinoma (PSCC) of the thyroid represents less than 1% of all primary thyroid malignancies and only a few cases have been reported in the world literature. Before diagnosing case as a primary squamous cell carcinoma of thyroid, possibility of other primary focuses of squamous cell carcinoma which can metastasize to thyroid must be excluded. The median survival after diagnosing case as a PSCC of thyroid is less than six months. Death is mainly due to persistent progression and local invasion by the tumor. Herein, we report case of thyroid cancer which was misdiagnosed as a papillary carcinoma by FNAC, but final histopathological examination after resection revealed diagnosis of squamous cell carcinoma of thyroid.

## 2. Case Report

**Figure 1:**
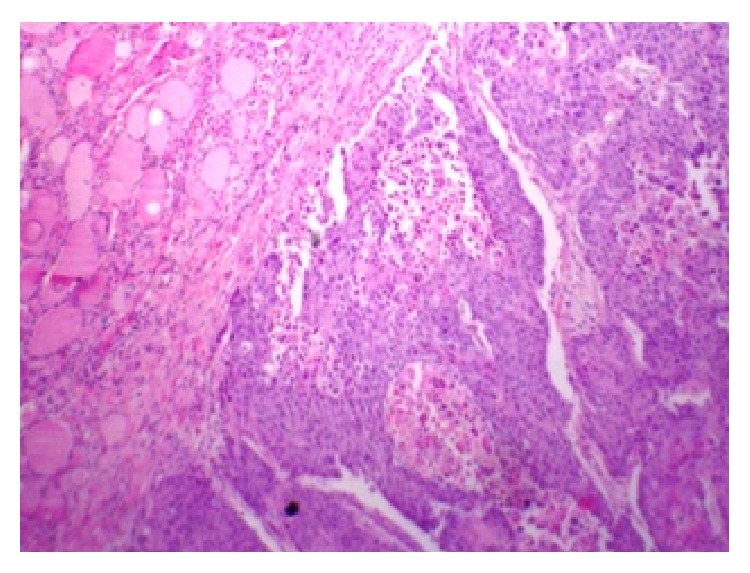
H/E × 100 section from the thyroid gland showing infiltrative squamous cell carcinoma of thyroid.

**Figure 2:**
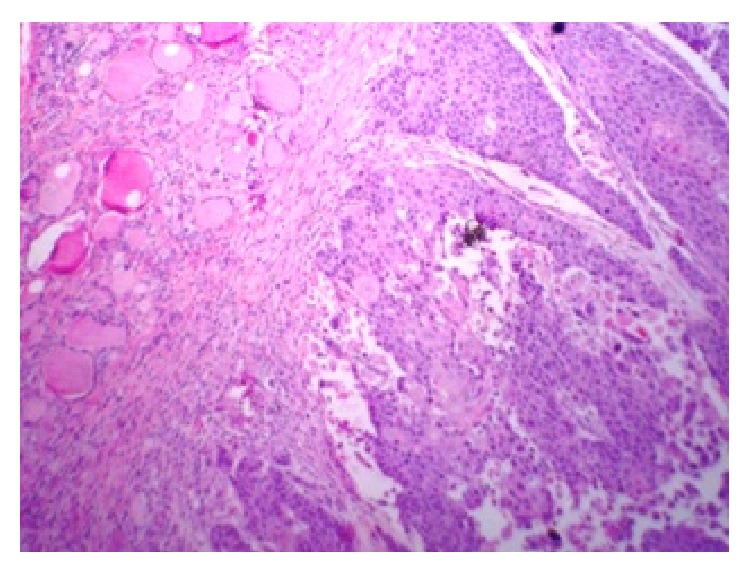
H/E × 100 section from the thyroid gland showing infiltrative squamous cell carcinoma of thyroid.

A 49-year-old lady presented with a mass in the left lower neck for 1 year. She had history of rapid growth of the same lesion over the last two weeks. There was no history of hyper/hypofunction of the thyroid. She complained of recent onset of pain associated with the swelling radiating to the back of the left ear, along with dysphagia. She had no addiction to tobacco or alcohol. On examination, the left lobe of the thyroid gland was enlarged (3 cm × 3 cm) with a palpable level IV cervical lymph node. Fine needle aspiration from the mass revealed papillary carcinoma of thyroid. Computed tomography (CT) scan of the neck showed a soft tissue density mass arising from the inferior aspect of left lobe of thyroid, extending up to the thoracic inlet and to the right side, with compression of oesophagus. Laryngoscopy was normal. Patient was planned for total thyroidectomy. Intraoperatively, the mass was adherent to soft tissue. A thyroidectomy and left sided radical neck dissection were done. Postoperatively, histopathological examination revealed a moderately to poorly differentiated squamous cell carcinoma of the thyroid with lymph node metastasis and invading adjacent soft tissue (Figures 1 and 2). Immunohistochemistry helped in making diagnosis. Tumor showed positivity for CK5/6, 7, 19 and p 63 as well. Tumor was negative for TTF- (thyroid transcription factor-) 1, thyroglobulin, calcitonin, p16, and CK20. Postoperatively, diligent search was made, if there was possible primary malignant lesion of squamous cell origin, causing metastasis to thyroid. Patient underwent thorough endoscopic studies, examination under anesthesia (along with tonsillectomy and directed biopsies), and imaging studies (CT and MRI scan of head and neck, CT scan of trunk, and PET-CT), but no possible origin for PSCC of thyroid could be identified. Patient was referred for chemotherapy and radiotherapy, still patient died within a year.

## 3. Discussion

As thyroid gland lacks squamous epithelium, PSCC of thyroid is a rare entity which represents less than 1% of thyroid carcinoma and only few cases have been reported in the literature [1]. It behaves like an anaplastic carcinoma with median survival approaching less than six months, often due to airway infiltration [2]. Secondary SCC is more common than primary one, either due to direct invasion or because of metastasis. PSCC of thyroid mainly affects female patients in their fifth or sixth decade of life and usually with history of goiter [3]. As in our case, patient may complain from rapid growth of the preexisting apparently benign swelling over recent past. Often tumor invades adjacent structures at the time of presentation, leading to difficulty in management. As we experienced, FNAC may not be able to diagnose the nature of lesion preoperatively and misdiagnosis because of FNAC may preclude the necessary preoperative workup in squamous cell carcinoma of thyroid.

Unique microscopic morphology (Figures 1 and 2), exclusion of other possible primary lesions, and help of immunohistochemistry make the final diagnosis of primary SCC of thyroid [3]. Anaplastic carcinoma, metastatic SCC, and carcinoma showing thymus-like differentiation (CASTLE) are other possible differential diagnoses for PSCC of thyroid. CASTLE shows less biological aggressive course along with positive immunoreactivity for CD5 [4, 5]. Exclusion of primary lesions in other organs is a paramount to differentiate between primary SCC and secondary SCC. In our patient no other possible primary focuses of squamous cell carcinoma were found with extensive investigation postoperatively.

As thyroid gland lacks squamous cell epithelium, how the squamous cell carcinoma originates in thyroid gland is a topic of great debate, and several theories have been suggested to explain this fact [3, 6]. Embryonic nest cell theory describes origin of squamous cell in thyroid gland, from remnant of thyroglossal duct [7]. Metaplastic theory suggests that it is due to chronic environmental stimulation which finally induces metaplasia in follicular epithelium [8]. Lastly, dedifferentiation theory postulates that existing (follicular, papillary, medullary, and anaplastic) thyroid carcinoma dedifferentiates into squamous cell carcinoma [9]. In the last few years because of few case reports, metaplastic theory is gaining recognition and some authors have observed PSCC of thyroid arising in the setting of lymphocytic thyroiditis, suggesting that squamous metaplasia of follicular cells is due to continuous stimulation [5].

Because of its rarity, the role and outcome of chemoradiation in management of PSCC of thyroid have not been properly studied, though many studies suggest that it is poorly responsive to either chemotherapy or radiotherapy [6, 10]. So the best treatment is early diagnosis and aggressive surgery with goal of achieving R0 resection, though it may be rarely possible. Our patient underwent surgery followed by chemoradiation; still she succumbed to death within a year; it underscores the aggressive natural history of this rare tumor.

## 4. Conclusion

Primary squamous cell carcinoma (PSCC) of thyroid is very rare and aggressive malignancy having median survival around six months. Pre-op FNAC may not be helpful in diagnosis. Often at the time of presentation PSCC of thyroid infiltrates surrounding structures. It is chemo- and radioresistant and surgery with R0 resection should be the goal whenever possible. After thorough clinical workup, primary focus must be excluded, before labeling case as a PSCC of thyroid.
